# Polygenic determinants in extremes of high-density lipoprotein cholesterol[S]

**DOI:** 10.1194/jlr.M079822

**Published:** 2017-09-04

**Authors:** Jacqueline S. Dron, Jian Wang, Cécile Low-Kam, Sumeet A. Khetarpal, John F. Robinson, Adam D. McIntyre, Matthew R. Ban, Henian Cao, David Rhainds, Marie-Pierre Dubé, Daniel J. Rader, Guillaume Lettre, Jean-Claude Tardif, Robert A. Hegele

**Affiliations:** Department of Biochemistry,* Schulich School of Medicine and Dentistry, Western University, London, Ontario, Canada; Robarts Research Institute,† Schulich School of Medicine and Dentistry, Western University, London, Ontario, Canada; Montréal Heart Institute et Faculté de Médecine,§ Université de Montréal, Montréal, Québec, Canada; Departments of Genetics and Medicine,‡ Perelman School of Medicine, University of Pennsylvania, Philadelphia, PA; Departments of Genetics, Medicine, and Pediatrics,‖ the Cardiovascular Institute, and the Institute for Translational Medicine and Therapeutics, Perelman School of Medicine, University of Pennsylvania, Philadelphia, PA; Department of Medicine,# Schulich School of Medicine and Dentistry, Western University, London, Ontario, Canada

**Keywords:** genetics, genomics, dyslipidemias, genes in lipid dysfunction, complex trait, rare variants, common variants, next-generation sequencing, polygenic risk score

## Abstract

HDL cholesterol (HDL-C) remains a superior biochemical predictor of CVD risk, but its genetic basis is incompletely defined. In patients with extreme HDL-C concentrations, we concurrently evaluated the contributions of multiple large- and small-effect genetic variants. In a discovery cohort of 255 unrelated lipid clinic patients with extreme HDL-C levels, we used a targeted next-generation sequencing panel to evaluate rare variants in known HDL metabolism genes, simultaneously with common variants bundled into a polygenic trait score. Two additional cohorts were used for validation and included 1,746 individuals from the Montréal Heart Institute Biobank and 1,048 individuals from the University of Pennsylvania. Findings were consistent between cohorts: we found rare heterozygous large-effect variants in 18.7% and 10.9% of low- and high-HDL-C patients, respectively. We also found common variant accumulation, indicated by extreme polygenic trait scores, in an additional 12.8% and 19.3% of overall cases of low- and high-HDL-C extremes, respectively. Thus, the genetic basis of extreme HDL-C concentrations encountered clinically is frequently polygenic, with contributions from both rare large-effect and common small-effect variants. Multiple types of genetic variants should be considered as contributing factors in patients with extreme dyslipidemia.

Despite apprehension over its direct causal role in atherogenesis and its value as a drug target (1), HDL cholesterol (HDL-C) remains a valid biochemical predictor of CVD risk (2–4). Understanding the full range of factors that determine plasma HDL-C concentrations, including genetics, still has relevance for epidemiology and risk projection (5). Furthermore, specific etiologies of extreme perturbations of HDL-C may have clinical importance in terms of diagnosis and directed therapies (1, 6).

Multiple genetic factors could be present in an individual, creating a polygenic network of influential determinants on HDL-C (7–9). These determinants include monogenic disorders (10, 11), such as extremely low or absent HDL-C due to rare homozygous mutations in *ABCA1*, *LCAT*, and *APOA1* (12–15), and extremely elevated HDL-C due to rare homozygous mutations in *CETP*, *LIPC*, and *SCARB1* (16–18). In contrast, the potential role of other genetic determinants in extreme HDL-C phenotypes, namely common SNPs (1), has not been systematically evaluated.

Polygenic factors, assessed by tallying SNPs with small phenotypic effects to derive “genetic risk scores” or “polygenic trait scores” (PTSs), contribute to numerous medical conditions, including coronary artery disease (19) and diabetes (20). Among dyslipidemias, polygenic factors play a substantial role in familial hypercholesterolemia (FH) (21), which was previously considered an archetypal “monogenic” disorder. For instance, in patients referred with extremely elevated LDL cholesterol (LDL-C), targeted next-generation sequencing demonstrated that ∼50% of individuals had rare heterozygous large-effect variants, whereas another ∼16% had an accumulation of common small-effect variants (i.e., SNPs) identified previously from genome-wide association studies (GWASs) as determinants of LDL-C (22). Although earlier sequencing experiments indicate that 11%–35% of patients with extremely low HDL-C and 5%–20% of patients with extremely high HDL-C have rare heterozygous large-effect variants (8, 23–28) driving the phenotypes, the proportion of such patients with excessive GWAS-identified SNPs, as quantified using PTSs, is unknown.

Here we used targeted next-generation sequencing to robustly characterize the genetic determinants influencing HDL-C levels in patients with extremely low and high HDL-C. This allowed us to concurrently evaluate the burden of rare large-effect variants and common small-effect GWAS variants, the latter bundled into a PTS. We saw that ∼30% of individuals at each HDL-C extreme had an identifiable genetic determinant, with the PTS explaining a marked increment above simple tallies of heterozygous large-effect variants. Our findings illustrate that both types of determinants are enriched in individuals with extremely high and low HDL-C levels.

## MATERIALS AND METHODS

### Study subjects

Two hundred and fifty-five unrelated patients with extreme HDL-C levels were selected for study from the Lipid Genetics Clinic at the London Health Sciences Centre, University Hospital (London, Ontario, Canada). Extremely low HDL-C was defined as ≤0.8 mmol/l (30.9 mg/dl) and ≤1.0 mmol/l (38.7 mg/dl) in males and females, respectively (N = 136). Extremely high HDL-C was defined as ≥1.4 mmol/l (54.1 mg/dl) and ≥1.8 mmol/l (69.6 mg/dl) in males and females, respectively (N = 119). These thresholds adhere closely to the top and bottom 10th percentiles of HDL-C levels in a North American population (29). The two patient exclusion criteria were triglyceride levels of ≥3.37 mmol/l (298.5 mg/dl) (as low HDL-C can simply be secondary to elevated triglycerides, which have their own distinct determinants) and diagnosis of clinical syndromes of extreme HDL-C (e.g., Tangier disease). All patients provided signed informed consent with approval from the Western University ethics review board (no. 07290E). As a reference group for the PTS analysis, the European subgroup of the 1000 Genomes Project (1KG; N = 503) was assumed to model the normal distribution of HDL-C levels among primarily normolipidemic individuals from the general population. A validation cohort from the Montréal Heart Institute (MHI) Biobank, ascertained as previously described (30), included individuals with extremely low HDL-C (N = 201), individuals with high HDL-C (N = 347), and normolipidemic controls (N = 1,198). A second validation cohort from the University of Pennsylvania (UPenn), ascertained as previously described (18, 31), included individuals with extremely low HDL-C (N = 349) as well as high HDL-C (N = 699). The studies in all clinic patients in this study were in compliance with the Declaration of Helsinki.

### DNA processing and sequencing

Genomic DNA from the Lipid Genetics Clinic were isolated with the Puregene® DNA Blood Kit (Gentra Systems, Qiagen Inc., Mississauga, Ontario, Canada) (cat. no. 158389). Samples were indexed and pooled with the Nextera® Rapid Capture Custom Enrichment Kit (cat. no. FC-140-1009) “LipidSeq” design (32). Genomic libraries of our enriched samples were sequenced with an Illumina MiSeq personal sequencer (Illumina, San Diego, CA). Sequencing and genotyping methods performed at the MHI Biobank (30) and UPenn (18) have been described in detail previously.

### Annotation of genetic variants

FASTQ files were generated for each patient and imported into CLC Bio Genomics Workbench (version 7.5; CLC Bio, Aarhus, Denmark) for read alignment against the human reference genome (build hg19), variant calling, and coverage statistics for targeted regions. Variants were annotated with customized ANNOVAR annotation scripts (33). Annotation methods performed at the MHI Biobank (30) and UPenn (18) have been described in detail previously.

### Identification of rare large-effect variants

Variants likely to produce extreme HDL-C phenotypes were identified using a specific set of criteria for both the Lipid Genetics Clinic and MHI cohorts. Definite causative variants previously reported as being phenotype-inducing in the Human Gene Mutation Database (http://www.hgmd.cf.ac.uk/ac/all.php) (34) were identified immediately. Of the remaining coding and splice-site variants, filters were applied for a minor allele frequency of <1% or missing according to the 1KG (http://browser.1000genomes.org/index.html) (35), Exome Sequencing Project (http://evs.gs.washington.edu/EVS/), and the Exome Aggregation Consortium (http://exac.broadinstitute.org/) (36) databases. Of the remaining coding variants, in silico predictions of deleterious or damaging outcomes were required for half of the available prediction tools, including Polymorphism Phenotyping (version 2; PolyPhen2; http://genetics.bwh.harvard.edu/pph2/) (37), Sorting Intolerant From Tolerant (SIFT; http://sift.jcvi.org/) (38), MutationTaster (http://www.mutationtaster.org/), or Combined Annotation Dependent Depletion (CADD; http://cadd.gs.washington.edu/score) (39). Concordance for pathogenic outcomes for Splicing Based Analysis of Variants (http://tools.genes.toronto.edu/) (40) and Automated Splice Site and Exon Definition Analyses (http://splice.uwo.ca/) (41) were necessary to identify splice-site variant candidates.

Of the variants meeting the above criteria, those within lipid-associated genes with direct (primary) and indirect (secondary) HDL-C-altering effects (supplemental Table S1) were designated as large-effect mutations; our use of the term “mutation” here is relatively colloquial, because it connotes the likely pathogenic nature of the large-effect variants.

It is important to note that because the UPenn cohort comes from an established on-going study (the UPenn High HDL-C Study), the criteria used in identifying large-effect variants differ slightly from what was considered here (18, 31). As such, carrier patients with rare large-effect variants found through that project were excluded from our study to ensure consistent results for subsequent analyses.

### Polygenic trait score

Between the discovery cohort and 1KG cohort, genotype data for 34 HDL-C-associated SNPs were available for study; these SNPs were selected from the most recent GWAS meta-analyses on blood lipids and lipoproteins, published by the Global Lipids Genetics Consortium (42). A PTS encompassing all available SNPs was calculated for all patients. In the interest of future application and usability, smaller SNP sets of 10 or fewer were tested and compared with the original set of 34; the aim was to select a smaller number of SNPs that were just as informative as was the full set of 34.

Scores were calculated by using a weighted approach; the number of HDL-C alleles associated with high HDL-C at a locus (0, 1, or 2) were summed and multiplied by the reported effect size for the high HDL-C-associated allele. The SNP products for each locus were totaled to provide the overall PTS for an individual. The underlying assumption when calculating the PTS was that each allele had an additive effect on their respective HDL-C phenotypes. Higher scores indicated that individuals carried a greater number of high HDL-C-associated alleles, and lower scores indicated that individuals carried fewer alleles associated with high HDL-C and therefore carried a greater number of low HDL-C-associated alleles.

### Statistical analysis

Differences between groups for mean PTS were evaluated with one-tailed, unpaired Wilcoxon rank-sum tests assuming unequal variances and are reported as the mean ± SD. Odds ratios (ORs) were derived using two-by-two contingency tables. Statistical analyses were conducted using SAS (version 9.3; SAS Institute, Cary, NC). Statistical significance was defined as *P* < 0.05 for the mean PTS comparisons and ORs.

## RESULTS

### Subject characteristics

Clinical and demographic information for the patients from the Lipid Genetics Clinic, the MHI Biobank, and UPenn are summarized in **Table 1**; the UPenn cohort has also been described in detail previously (31). In the Lipid Genetics Clinic, the majority of individuals reported European descent, with <3% of individuals reporting Asian or African ancestry. In the MHI Biobank cohort, all individuals are of French-Canadian ancestry.

**TABLE 1.** Demographics of extreme HDL-C patient cohorts

	Lipid Genetics Clinic				Montréal Heart Institute Biobank				University of Pennsylvania			
	Low HDL-C		High HDL-C		Low HDL-C		High HDL-C		Low HDL-C		High HDL-C	
	Males	Females	Males	Females	Males	Females	Males	Females	Males	Females	Males	Females
N	90	46	60	59	131	70	280	67	202	147	217	482
Age	48.1 ± 16.8*^a^*	45.4 ± 12.5*^a^*	58.5 ± 14.2	58.6 ± 10.5	64.4 ± 10.4	68.9 ± 8.4	65.6 ± 10.1	71.2 ± 7.2	56.0 ± 12.2	53.2 ± 15.0	58.7 ± 14.9	58.2 ± 11.7
BMI	29.0 ± 5.6*^a^*	28.8 ± 6.0*^a^*	26.5 ± 3.7	25.3 ± 3.5*^a^*	31.0 ± 5.2*^a^*	31.4 ± 6.4*^a^*	26.9 ± 4.5*^a^*	26.4 ± 6.0*^a^*	32.4 ± 5.0*^a^*	34.5 ± 7.4*^a^*	29.0 ± 5.0*^a^*	27.2 ± 7.0*^a^*
TC	4.2 ± 1.4	5.8 ± 2.3	5.7 ± 1.4	6.9 ± 1.5	3.4 ± 1.1	3.7 ± 1.0	4.5 ± 1.0*^a^*	5.2 ± 1.1	4.0 ± 1.1	4.5 ± 1.3	6.5 ± 1.6	6.4 ± 1.2
HDL-C	0.6 ± 0.2	0.8 ± 0.2	2.1 ± 0.5	2.7 ± 0.7	0.7 ± 0.1	0.9 ± 0.1	1.7 ± 0.2	2.1 ± 0.3	0.8 ± 0.2	0.9 ± 0.2	2.5 ± 0.5	2.9 ± 0.5
LDL-C	2.7 ± 1.3	4.0 ± 2.3	3.2 ± 1.4	3.7 ± 1.5	2.2 ± 1.0	2.2 ± 0.9	2.4 ± 0.9*^a^*	2.5 ± 0.9	2.7 ± 1.3	3.2 ± 1.7	3.0 ± 1.2	3.6 ± 2.0
TG	2.2 ± 1.3	2.0 ± 1.1	1.0 ± 0.5	1.2 ± 0.6	2.2 ± 0.7	2.3 ± 0.7	1.4 ± 0.6	1.3 ± 0.5	1.8 ± 0.7	1.6 ± 0.6	0.9 ± 0.4	0.9 ± 0.4
CVD Hx (%)	45.2*^a^*	21.9*^a^*	29.8*^a^*	16.7*^a^*	60.3	67.1	40.7	31.3	11.9*^a^*	12.9*^a^*	6.0*^a^*	4.1*^a^*

Values are indicative of the mean ± SD. Lipid values are in millimoles per liter. CVD Hx, CVD history; TC, total cholesterol.

a An incomplete dataset was used for these calculations.

### Rare large-effect variants in HDL-C-altering genes

**Fig. 1.**
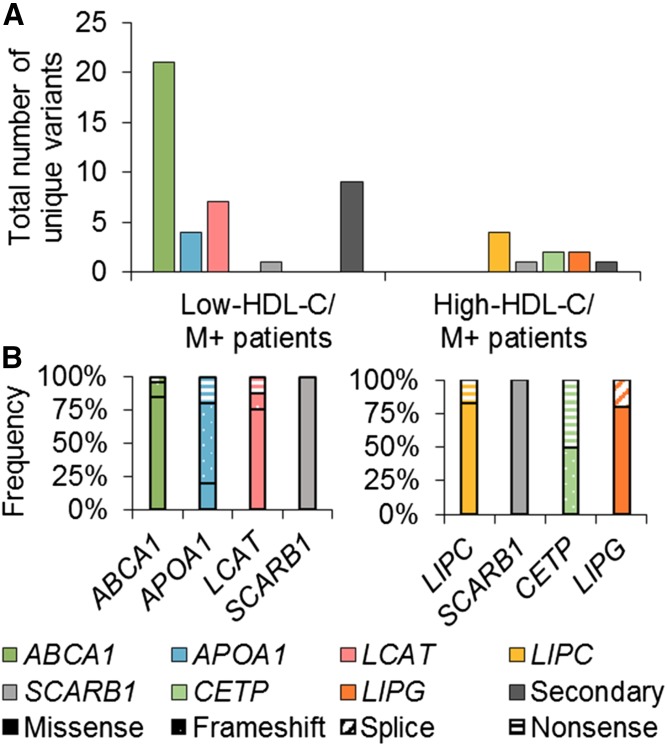
Summary of rare large-effect variant types in the Lipid Genetics Clinic cohort. Forty-three unique variants were identified in primary genes, and 10 unique variants were identified in secondary genes; there were 68 variants total. A: Total number of unique variants per gene per patient group. B: Frequency of variant type for each unique variant for the low-HDL-C M+ patients (left) and high-HDL-C M+ patients (right).

Considering the primary genes associated with low HDL-C (i.e., *ABCA1*, *APOA1*, *LCAT*) and high HDL-C (i.e., *LIPC*, *SCARB1*, *CETP*, and *LIPG*), we identified 43 unique large-effect variants that are either likely or definite causes of either extreme (**Fig. 1A** and supplemental Table S2). When considering variant types, 72.1% were missense, 4.7% were splicing, 14.0% were frameshift, and 9.3% were nonsense variants (Fig. 1B). One individual was homozygous for *ABCA1* p.G851R, and one individual was a compound heterozygote for *ABCA1* p.W590C and p.W590L. A single individual carried rare heterozygous variants in both a low and a high HDL-C-associated gene, that is, *ABCA1* and *SCARB1*, and presented with a low HDL-C phenotype.

Only a few large-effect variants were identified in secondary HDL-C-altering genes (supplemental Table S2). In nine low-HDL-C patients, missense variants were identified in *LPL*, *APOA5*, *LMF1*, *GPD1*, and *APOE*. In two high-HDL-C patients, the same splicing variant was identified in *APOC3*. All variants in the secondary genes were heterozygous.

Overall, 30.1% and 12.6% of low- and high-HDL-C patients, respectively, carried at least one variant that could explain their phenotypes and were labeled “mutation positive” (M+). Conversely, patients who were noncarriers were labeled “mutation negative” (M−). Patients were grouped by HDL-C phenotype and mutation status (i.e., M+ or M−) (supplemental Fig. S1).

In the MHI validation cohort, 10.9% of patients with low HDL-C and 10.4% of patients with high HDL-C carried large-effect variants, respectively. The same distinctions between phenotype and M+ or M− status were applied. In the UPenn validation cohort, because different criteria were used in large-effect variant identification, only M− patients were considered for this study.

### Measuring accumulation of common small-effect variants using a polygenic trait score

A set of nine SNPs produced results that most closely matched the results from the original 34-SNP score and was used as the primary score in this study. The nine SNPs were in linkage equilibrium and showed significant primary associations with plasma levels of HDL-C; some of the loci were previously implicated either directly or indirectly to HDL metabolism (**Table 2**). Each SNP was selected on the basis of their reported β coefficients, or “effect sizes,” reported *P* values, and frequency within the general population. The allele associated with higher HDL-C levels was taken as the primary variable.

**TABLE 2.** Nine SNPs used in polygenic trait score

Chr:Position	rsID	Gene	High HDL-C-Associated Allele	Effect Size	Relation with HDL-C or HDL Metabolism
1:182199750	rs1689800	*ZNF648*	A	0.034	Mechanism underlying association is poorly characterized.
1:230159944	rs4846914	*GALNT2*	A	0.048	Recently confirmed as an important determinant of HDL-C (43)
9:104902020	rs1883025	*ABCA1*	C	0.07	Causative gene for Tangier disease (6)
12:109562388	rs7134594	*MVK*	T	0.035	*MVK* encodes mevalonate kinase, which is involved in biosynthesis of cholesterol and isoprenoids (44), although the closely linked *MMAB* gene encoding cob(I)alamin adenosyltransferase may actually underlie the HDL-C association at this locus (45).
12:124777047	rs838880	*SCARB1*	C	0.048	Causative gene for scavenger receptor B1 deficiency
15:58391167	rs1532085	*LIPC*	A	0.107	Causative gene for hepatic lipase deficiency
16:56959412	rs3764261	*CETP*	A	0.241	Causative gene for cholesteryl ester transfer protein deficiency
16:81501185	rs2925979	*CMIP*	C	0.035	Mechanism underlying association is poorly characterized.
19:8368312	rs7255436	*ANGPTL4*	A	0.032	Regulates LPL with reciprocal effects on triglycerides and HDL-C (46)

SNP information from this table has been extracted from (42). Chr, chromosome; rsID, reference SNP cluster ID (accession number).

**Fig. 2.**
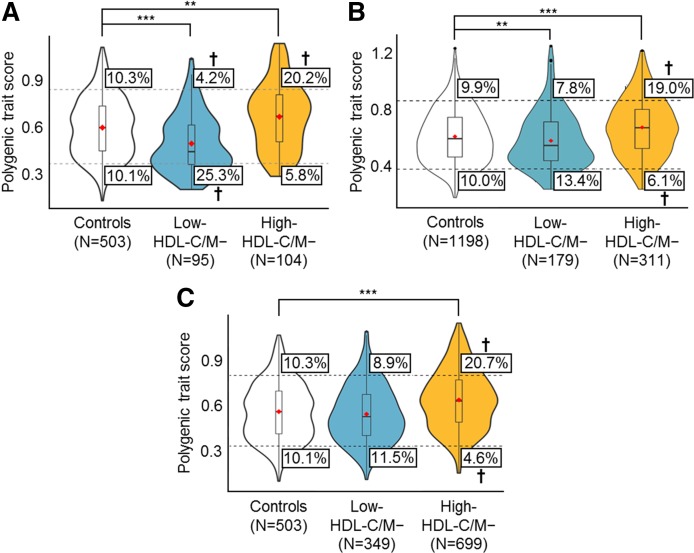
PTS analysis for low- and high-HDL-C/M− patients. Violin plots (similar to box plots, except that they also show the probability density of the data at different values) illustrate the distribution of polygenic scores for individuals in the control, low-HDL-C/M−, and high-HDL-C/M− groups in the Lipid Genetics Clinic cohort (A), the MHI Biobank cohort (B), and UPenn cohort (C). Red diamonds mark the mean PTS of the group. The total area of a single plot represents 100% of individuals in that group; wider sections of each plot represent an increased number of individuals with scores at that point, and narrower sections represent a decreased number of individuals. The top dashed line and bottom dashed line represent the threshold for the top 10th and bottom 10th percentiles of PTSs in the control population, respectively. The boxes indicate the percentage of individuals falling above or below the percentile thresholds. ^†^Percentile groups with significant ORs (Tables 3, 4); **P < 0.01; ***P < 0.0001.

The PTS results for the Lipid Genetics Clinic cohort were analyzed by phenotype and mutation status rather than on an individual level and were visualized with violin plots (**Fig. 2A**), which illustrate the distribution of PTSs within reference individuals (i.e., the European subgroup of the 1KG population) and low- or high-HDL-C patients. Neither the low- nor high-HDL-C/M+ group had a mean PTS that was significantly different from the reference population.

**Fig. 3.**
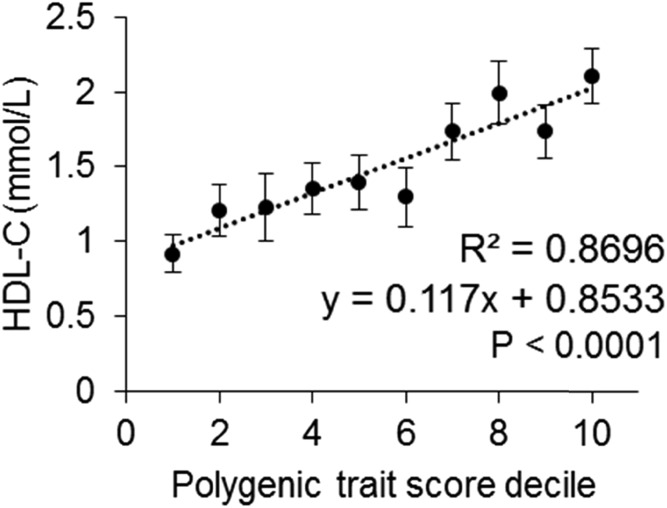
Mean HDL-C level in low- and high-HDL-C/M− patients from the Lipid Genetics Clinic by PTS decile. There is a strong linear relationship between increasing PTSs and HDL-C levels, as is indicated by the *R*^2^ value of 0.8696 (*P* < 0.0001). Vertical bars indicate standard errors of the mean.

However, the mean PTSs for the low-HDL-C/M− patients (0.48 ± 0.18, unpaired Wilcoxon rank-sum, *P* < 0.0001) and the high-HDL-C/M− patients (0.65 ± 0.21, unpaired Wilcoxon rank-sum, *P* = 0.0015) were significantly less than and greater than the reference population (0.58 ± 0.19), respectively. In addition, 25.3% of low-HDL-C/M− patients fell below the bottom 10th percentile in comparison with 10.1% of the reference population (OR: 3.00 [95% CI: 1.67–5.35], *P* < 0.0001), whereas 20.2% of high-HDL-C/M− patients fell above the top 10th percentile in comparison with 10.3% of the reference population (OR: 2.19 [95% CI: 1.21–3.96], *P* = 0.006). A primarily polygenic basis for extreme HDL-C was considered for M− patients with an “extreme” PTS, below the bottom 10th or above the top 10th percentile for low- or high-HDL-C phenotypes, respectively (supplemental Fig. S2). When patients are grouped by PTS decile, there is a strong linear relationship between increasing PTS and HDL-C level (**Fig. 3**).

### External validation of polygenic trait score

Results from the MHI validation cohort were similar to those of the Lipid Genetics Clinic cohort. Groups with a mean PTS that was significantly different from the reference population (0.58 ± 0.19) were the low-HDL-C/M− patients (0.55 ± 0.20, *P* = 0.007) and the high-HDL-C/M− patients (0.64 ± 0.20, *P* < 0.0001) (Fig. 2B). Additionally, only the high-HDL-C/M− patients showed a significant OR: 19.0% of patients fell above the top 10th percentile in comparison with 9.9% of reference individuals (OR: 2.12 [95% CI: 1.48–3.02], *P* < 0.0001) (**Tables 3**, **4**). From the UPenn validation cohort, only the high-HDL-C/M− individuals (0.66 ± 0.20, *P* < 0.0001) had a mean PTS that was significantly different from that of the reference population (0.58 ± 0.19) (Fig. 2C). Similarly, only the high-HDL-C/M− patients showed a significant OR: 20.7% of patients fell above the top 10th percentile in comparison with 10.3% of reference individuals (OR: 2.27 [95% CI: 1.59–3.24], *P* < 0.0001) (Tables 3, 4).

**TABLE 3.** PTS comparison of patients based on low HDL-C levels and mutation status

	Top 10th Percentile of PTS				Bottom 10th Percentile of PTS			
	Control	M+	M−	OR (95% CI, *P*)	Control	M+	M−	OR (95% CI, *P*)
Lipid Genetics Clinic	52/503	4/41	4/95	M+: 0.94 (0.27–2.90, 0.583)	51/503	8/41	24/95	M+: 2.15 (0.86–5.19, 0.063)
				**M**−**: 0.38 (0.11**–**1.13, 0.038)**				**M**−**: 3.00 (1.67**–**5.35, <0.0001)**
MHI Biobank	119/1,198	3/22	14/179	M+: 1.43 (0.33–5.21, 0.380)	120/1,198	1/22	24/179	M+: 0.43 (0.02–3.03, 0.341)
				M−: 0.77 (0.41–1.41, 0.228)				M−: 1.39 (0.85–2.27, 0.107)
UPenn	52/503	NA	31/349	M+: NA	51/503	NA	40/349	M+: NA
				M−: 0.85 (0.52–1.38, 0.280)				M−: 1.15 (0.72–1.82, 0.307)
Overall	171/1,701*^a^*	7/63	49/623	M+: 1.12 (0.46–2.60, 0.455)	171/1,701*^a^*	9/63	88/623	M+: 1.49 (0.67–3.20, 0.186)
				M−: 0.76 (0.54–1.08, 0.063)				**M**−**: 1.47 (1.11–1.96, <0.01)**

Whole numbers represent patient counts, with the numerator indicating the number of individuals with a PTS in the top 10th or bottom 10th percentile and the denominator indicating the total number of individuals in the patient subgroup. ORs were calculated with a confidence level of 95%. Left-tailed and right-tailed tests of significance were considered for the top 10th and bottom 10th percentiles, respectively. NA, not available. Boldface type indicates statistically significant ORs.

a The 1KG group was counted only once, given that it was used in both the Lipid Genetics Clinic and UPenn cohorts.

**TABLE 4.** PTS comparison of patients based on high HDL-C levels and mutation status

	Top 10th Percentile of PTS				Bottom 10th Percentile of PTS			
	Control	M+	M−	OR (95% CI, *P*)	Control	M+	M−	OR (95% CI, *P*)
Lipid Genetics Clinic	52/503	2/15	21/104	M+: 1.33 (0.20–6.47, 0.476)	51/503	0/15	6/104	M+: 0 (0–3.11, 0.207)
				**M**−**: 2.19 (1.21**–**3.96, <0.01)**				M−: 0.54 (0.20–1.36, 0.110)
MHI Biobank	119/1,198	4/36	59/311	M+: 1.13 (0.33–3.44, 0.490)	120/1,198	2/36	19/311	M+: 0.53 (0.09–2.29, 0.291)
				**M**−**: 2.12 (1.49**–**3.03, <0.0001)**				**M**−**: 0.59 (0.34**–**0.99, 0.019)**
UPenn	52/503	NA	145/699	M+: NA	51/503	NA	32/699	M+: NA
				**M−: 2.27 (1.59**–**3.24, <0.0001)**				**M**−**: 0.43 (0.26**–**0.69, <0.0001)**
Overall	171/1,701*^a^*	6/51	225/1,114	M+: 1.19 (0.45–2.97, 0.412)	171/1,701*^a^*	2/51	57/1,114	M+: 0.37 (0.6–1.55, 0.106)
				**M−: 2.27 (1.82**–**2.83, <0.0001)**				**M−: 0.48 (0.35–0.67, <0.0001)**

Whole numbers represent patient counts, with the numerator indicating the number of individuals with a PTS in the top 10th or bottom 10th percentile and the denominator indicating the total number of individuals in the patient subgroup. ORs were calculated with a confidence level of 95%. Right-tailed and left-tailed tests of significance were considered for the top 10th and bottom 10th percentiles, respectively. Boldface type indicates statistically significant ORs.

a The 1KG group was counted only once, given that it was used in both the Lipid Genetics Clinic and UPenn cohorts.

## DISCUSSION

We report a polygenic trait score for HDL-C that expands the proportion of individuals with extreme levels that can be explained genetically. In our subjects, we first confirmed an excess of rare heterozygous large-effect variants in *ABCA1*, *LCAT*, and *APOA1*, and in *CETP*, *LIPC*, *LIPG*, and *SCARB1* among individuals with extremely low and high HDL-C, respectively. Overall, 18.7% of low- and 10.9% of high-HDL-C patients carried at least one such variant. Then, among remaining individuals with extreme levels and without such rare variants, we showed an ∼1.5- to 2-fold increased risk of having an extreme PTS due to multiple common small-effect variants known to influence HDL-C levels from earlier GWAS (*P* < 0.01 for each extreme). Overall, 12.8% of low-HDL-C patients and 19.3% of high-HDL-C patients had an extreme PTS. Cumulatively, >30% of individuals had either a rare large-effect variant or a bundle of common small-effect variants associated with their respective extreme HDL-C phenotype. Our study highlights the importance of polygenic effects as determinants of extreme HDL-C and reinforces the polygenic nature of this complex trait.

In Lipid Genetics Clinic patients, 47.7% and 30.2% of low- and high-HDL-C patients, respectively, had identifiable genetic contributors to their extreme phenotypes. The prevalence of M+ patients in the low-HDL-C subgroup was higher than was the proportion of M+ patients in the MHI cohort, perhaps reflecting ascertainment bias. Mean HDL-C levels were markedly lower in the clinically ascertained low-HDL-C extreme subgroup than in the MHI and UPenn cohorts; rare large-effect variants may be more important determinants of the phenotype. Furthermore, there was no excess of extreme PTSs among M+ individuals with both extremes of HDL-C in both study cohorts, suggesting that when a large-effect variant was present, it was the main determinant of the extreme HDL-C phenotype, overriding small polygenic effects.

In contrast, among clinically ascertained M− individuals with low HDL-C, a large excess had low PTSs (OR: 3.00 [95% CI: 1.67–5.35], *P* < 0.0001). There were nonsignificant trends to low PTSs among M− patients from MHI and UPenn, leading to an excess risk for a low PTS in the overall low-HDL-C/M− sample (OR: 1.47 [95% CI: 1.11–1.96], *P* < 0.01). This pattern was mirrored by respective deficits of high PTSs in the same subgroups (Table 3). This demonstrates that individuals with low HDL-C and no large-effect variants have a significant polygenic contribution of small-effect variants. In the Lipid Genetics Clinic, MHI, and UPenn cohorts, among clinically ascertained M− individuals with high HDL-C, many had a high PTS (overall OR: 2.27 [95% CI: 1.82–2.83], *P* < 0.0001). This pattern was mirrored by deficits of low PTSs in the same subgroups (Table 4). This demonstrates that among individuals with high HDL-C and no large-effect variants, there was a significant polygenic contribution from small-effect variants.

We also found that M+ individuals with the respective phenotype and concurrent excess PTS did not have HDL-C levels that were significantly different from those of M+ individuals with normal PTSs (data not shown). This suggests that rare large-effect variants and polygenic determinants are independent and, when present together, are not necessarily additive: rare large-effect variants appear to predominantly determine the HDL-C phenotype. This contrasts with conclusions derived from a whole-genome sequence analysis of individuals with less extreme phenotypes, in whom common variants were determined to be the predominant determinants of HDL-C (47). Of course, our cohorts were still relatively small: a possible additive or synergistic relationship between large- and small-effect variants will require evaluation in much larger samples of such extreme individuals.

Application of PTSs, or “genetic risk scores,” is becoming popular in the area of cardiovascular health and related complex traits (48). Mendelian randomization studies have previously evaluated genetic risk scores to infer a causal role of HDL-C in CVD outcomes (49). However, until now there has been minimal to no evaluation of polygenic scores in individuals selected for extremes of HDL-C levels.

Among extreme dyslipidemias, PTSs have been well studied in cohorts of patients with extremely high LDL-C levels, particularly FH. In fact, the genetic architecture is analogous among individuals with FH and those with extreme HDL-C studied here. For instance, among clinically ascertained individuals with likely FH, 50%–80% have a rare heterozygous large-effect variant in *LDLR*, *APOB*, or *PCSK9*, whereas another 15%–20% have an extreme PTS comprising small-effect SNPs for LDL-C (21, 22). The exact proportions of individuals with large- and small-effect variants differ in our cohorts with extreme HDL-C levels, but the overall pattern of genetic contributors to both complex lipoprotein traits is similar. One possible difference is that syndromic FH was intentionally enriched in the extreme LDL-C studies, whereas we excluded patients with known clinical syndromes of extreme HDL-C.

Also, for LDL-C, only individuals with extremely high levels are typically studied. In contrast, our current study assessed individuals with both very low and very high HDL-C extremes. The fact that our PTS was directionally associated with both extremes of HDL-C (i.e., excessive high and low PTS among individuals with high and low HDL-C phenotypes, respectively) indicates that this score applies bidirectionally for HDL-C and is thus relatively unique among such scores evaluated for most quantitative metabolic traits.

There may be clinical relevance in knowing the genetic basis of a patient’s extreme HDL-C level. For instance, in patients with high LDL-C, the CVD risk compared to normolipidemic individuals was ∼22-fold higher in those who carried a rare heterozygous large-effect variant in comparison with ∼6-fold higher among those who did not (50). Although polygenic effects were not evaluated, extreme LDL-C in at least some individuals in the latter subgroup likely had a polygenic basis. Although both groups are at high risk having such patient substrata, it generates hypotheses for different interventions under the framework of precision medicine. For instance, prospective randomized studies may show that among individuals with extremely high LDL-C, carriers of a rare variant may benefit relatively more from certain treatments, such as PCSK9 inhibitors, than might individuals with a polygenic basis (51). By analogy, individuals with extremely low HDL-C who carry a rare variant versus those who have a high polygenic burden can be studied to determine whether there are differential effects of therapies targeted toward raising HDL-C (52).

This study has some limitations. First, patient ascertainment differed among the three cohorts: Lipid Genetics Clinic patients were referred because of abnormal lipid profiles; MHI Biobank participants were recruited on the basis of cardiovascular health; and though UPenn patients also came from lipid referrals, there was more focus on high-HDL-C phenotypes. This may explain why the low-HDL-C patients from the discovery cohort had a greater burden of rare variants: these individuals’ HDL-C phenotypes were more pronounced and perhaps more likely to have a genetic basis. In contrast, because CVD was of primary interest at the MHI, abnormal HDL-C profiles were less extreme and perhaps more often secondary to other, nongenetic health issues. Testing the PTS in additional cohorts with more closely matched patient-ascertainment parameters would not only increase the power of our study but also alleviate these biases. Second, application of the PTS assumes that each allele has a linearly additive effect, with no epistatic interactions. Modeling epistasis could improve PTS accuracy and comprehension. Third, the PTS was tested largely in individuals of European ancestry and may not be generalizable to other ethnic groups. Also, we did not evaluate other factors, such as epigenetic regulators or large copy-number variations, as possible explanations for their extreme phenotypes. Additionally, some important variants may have been overlooked, because only specific known genes associated with HDL-C were screened and only a subset of HDL-C-associated SNPs were considered; this could have led to a skew in the percentage of M+ patients or patients with an extreme accumulation of polygenic SNPs. Finally, given that low-pass whole-genome sequencing was used to genetically characterize participants from the MHI Biobank, it is possible that rare variants in the HDL-C candidate genes may have been missed. Despite these limitations, we have for the first time demonstrated the genetic complexity underlying extreme HDL-C phenotypes by considering both rare variants and the accumulation of common SNPs simultaneously.

In summary, we concurrently detected both rare large-effect and common small-effect variants using our next-generation sequencing platform. In patients with both low- and high- HDL-C extremes, we confirmed the enrichment of rare large-effect variants, and simultaneously detected individuals with extreme PTSs. This substantially expanded the number of individuals with a genetic basis for their phenotype: about one-sixth of patients with extreme HDL-C had an extreme PTS. Loci for rare and common variants contributing to extreme HDL-C levels encode products acting at all stages of the HDL lifecycle; we suggest that both rare and common variants be considered concurrently for understanding extreme HDL-C. The large proportion of individuals still unaccounted for can be studied for additional mechanisms, such as possible new genes, gene-gene or gene-environment interactions, and nonmendelian influences, including mitochondrial or epigenetic effects. In addition to acquiring a more complete genetic picture of patients with extreme dyslipidemia, stratifying them genetically may help evaluate interindividual differences in their clinical course or responses to interventions.

## Supplementary Material

Supplemental Data
