# Supplementary material for: Polygenic determinants in extremes of high-density lipoprotein cholesterol
Source: J Lipid Res. 2017 Sep 4;58(11):2162–70. doi: 10.1194/jlr.M079822 (PMC5665671; doi:10.1194/jlr.M079822)
Supplement: Supplemental Data [file supp_58_11_2162__index.html]

Polygenic determinants in extremes of high-density lipoprotein cholesterol — Polygenic determinants in extremes of high-density lipoprotein cholesterol — Supplemental Data 

# Polygenic determinants in extremes of high-density lipoprotein cholesterol

## Supplemental Data

- Supplemental information (.pdf, 266 KB) - Supplemental information
