# Supplementary material for: Polygenic determinants in extremes of high-density lipoprotein cholesterol
Source: J Lipid Res. 2017 Sep 4;58(11):2162–70. doi: 10.1194/jlr.M079822 (PMC5665671; doi:10.1194/jlr.M079822)
Supplement: Supplemental Data [file 10.1194_M079822_jlr.M079822-1.pdf]

SUPPLEMENTAL FIGURES

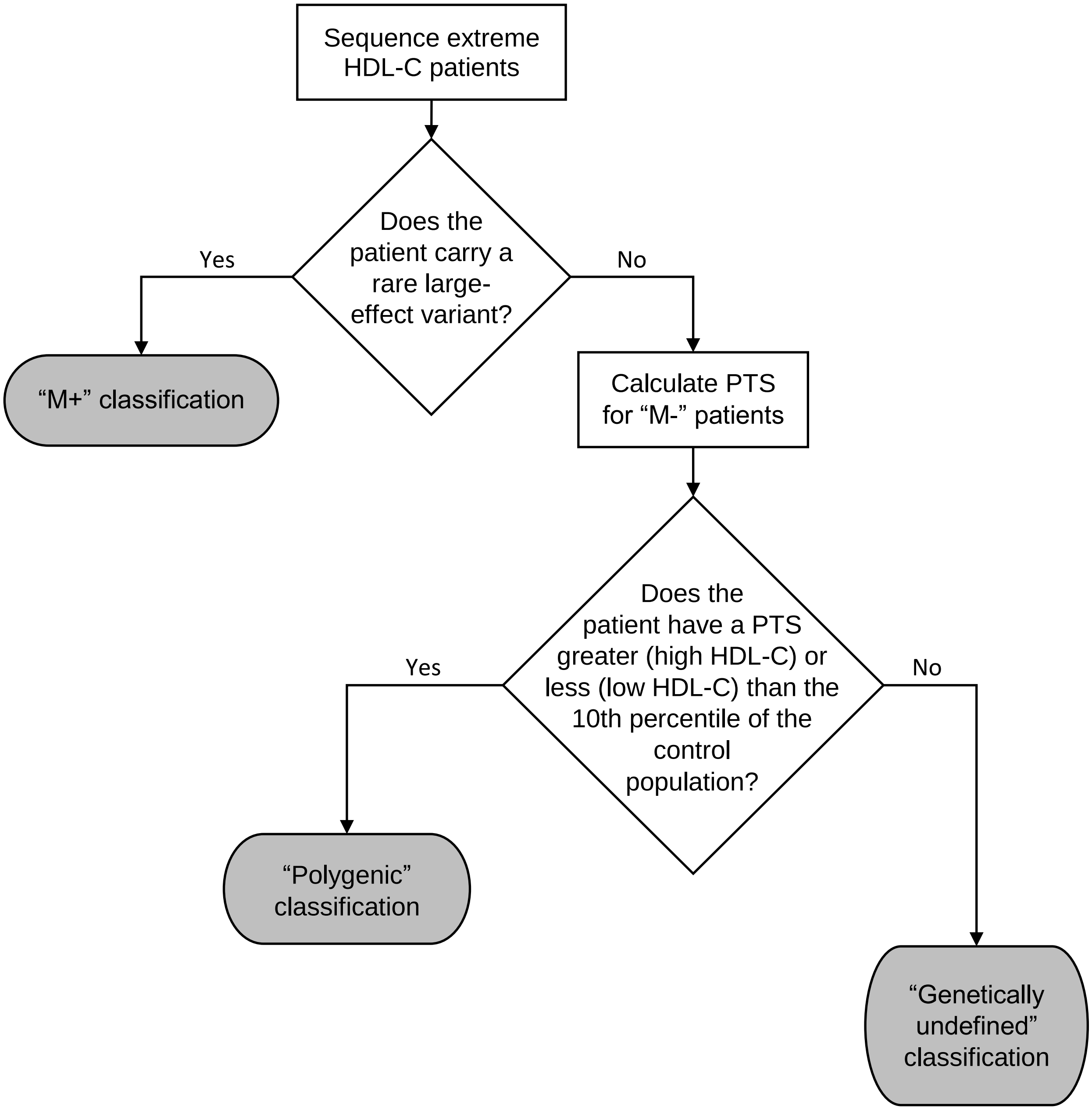

Supplemental Figure S1. Process to genetically classify extreme HDL-C patients. HDL-C = HDL cholesterol; M+ = mutation positive; M- = mutation negative; PTS = polygenic trait score.

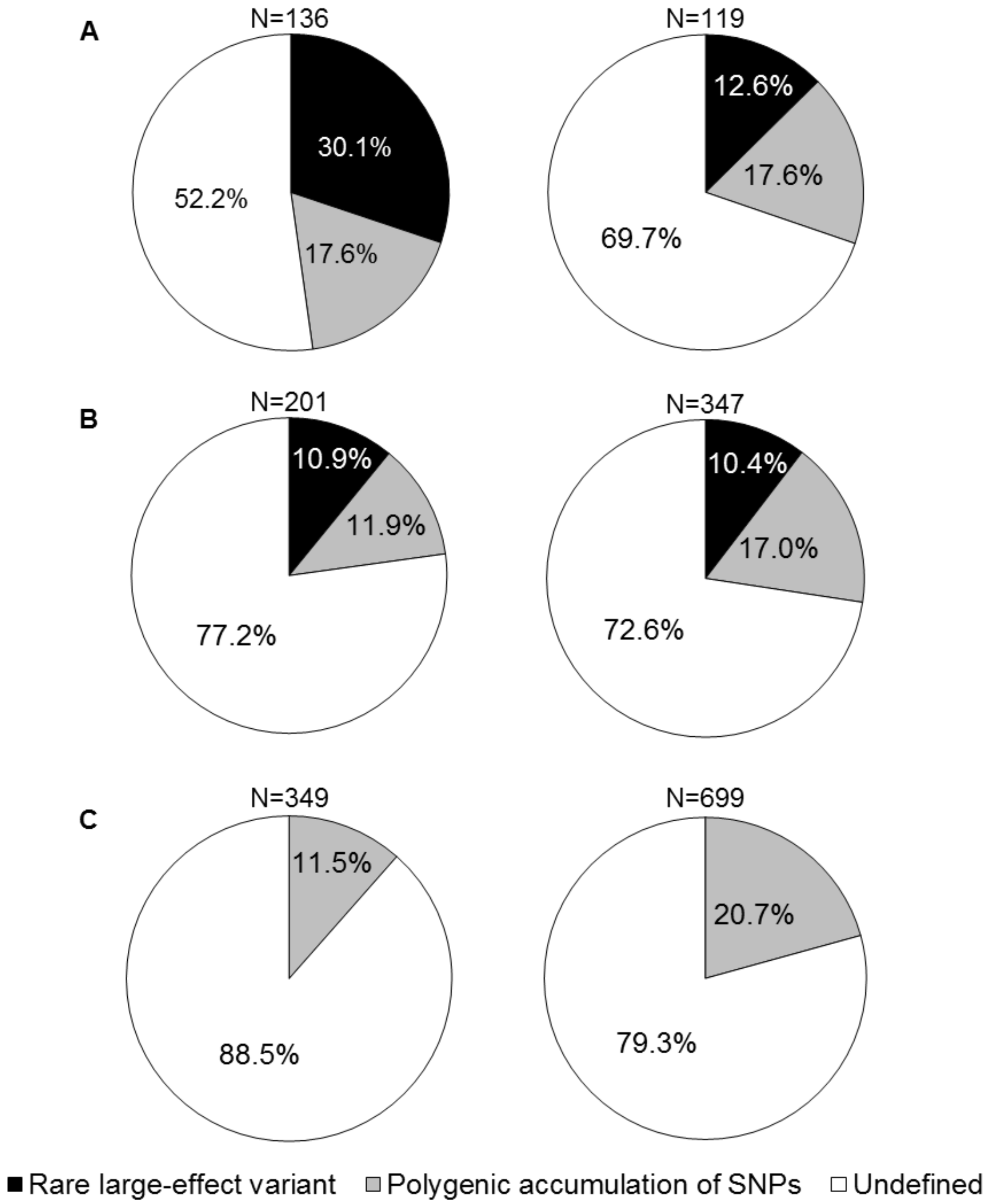

Supplemental Figure S2. Genetic determinants of extreme HDL-C phenotypes. Percentages were determined from the low (left) and high (right) HDL-C patients from the **A:** Lipid Genetics Clinic (N=255), **B:** the MHI Biobank cohort (N=548), and **C:** UPenn cohort (N=1,048). It must be noted that the UPenn cohort only consists of M- individuals, meaning that none of the individuals carried rare large-effect variants. HDL-C = HDL cholesterol; M- = mutation negative; MHI = Montréal Heart Institute; SNP = single nucleotide polymorphism; UPenn = University of Pennsylvania.

SUPPLEMENTAL TABLES

Supplemental Table S1. Genes with direct (primary) and indirect (secondary) influences on HDL-C levels

Influence on HDL-C	Phenotype	Gene	Related disorder
Primary	Low HDL-C	*ABCA1*	Tangier disease
		APOA1	Apolipoprotein A-I deficiency
		LCAT	Familial LCAT deficiency
	High HDL-C	*LIPC*	Hepatic lipase deficiency
		SCARB1	Scavenger receptor B1 deficiency
		CETP	Cholesteryl ester transfer protein deficiency
		LIPG	Hyperalphalipoproteinemia
Secondary	Low TG	*APOC3*	Apolipoprotein C-III deficiency
	High TG	*LPL*	Lipoprotein lipase deficiency
		APOA2	Apolipoprotein C-II deficiency
		APOA5	Apolipoprotein A-V deficiency
		LMF1	Lipase maturation factor deficiency
		GPIHBPI	Severe hypertriglyceridemia
		GPD1	Infantile hypertriglyceridemia
		APOE	Dysbetalipoproteinemia

Genes of interest were selected based on reporting by Johansen CT et al. *J Lipid Res* (2014). Abbreviations: HDL-C = HDL cholesterol.

Supplemental Table S2. Summary of likely and definite causative mutations producing extreme HDL-C phenotypes

Gene	Nucleotide change	Amino acid change	Mutation type	Allele count	HGMD	Prediction outcomes[a]	Phenotype
Primary							
ABCA1	c.-357G>A	p.M1ext-119[b]	Missense	1		7.3 | NA | NA | NA	Low HDL-C
ABCA1	c.103A>G	p.I35V	Missense	1		22.9 | Benign | Damaging | Damaging	Low HDL-C
ABCA1	c.206G>T	p.W69L	Missense	2		26.3 | Probably damaging | Damaging | Damaging	Low HDL-C
ABCA1	c.208delG	p.V70fsX53	Frameshift	2		22.9 | NA | NA | NA	Low HDL-C
ABCA1	c.688C>T	p.R230C	Missense	1	Yes	19.22 | Benign | Tolerated | Tolerated	Low HDL-C
ABCA1	c.1660T>C	p.Y554H	Missense	1		27.5 | Possibly damaging | Damaging | Damaging	Low HDL-C
ABCA1	c.1770G>C	p.W590C	Missense	1	Yes	24.7 | Possibly damaging | Damaging | Damaging	Low HDL-C
ABCA1	c.1769G>T	p.W590L	Missense	1	Yes	24.7 | Possibly damaging | Tolerated | Damaging	Low HDL-C
ABCA1	c.2270T>C	p.L757P	Missense	2		28 | Probably damaging | Damaging | Damaging	Low HDL-C

ABCA1	c.2328G>C	p.K776N	Missense	2	Yes	27.6 | Probably damaging | Damaging | Damaging	Low HDL-C
ABCA1	c.2551G>A	p.G851R	Missense	2		34 | Probably damaging | Damaging | Damaging	Low HDL-C
ABCA1	c.2819C>T	p.T940M	Missense	1	Yes	32 | Probably damaging | Damaging | Damaging	Low HDL-C
ABCA1	c.3191A>G	p.D1064G	Missense	1		29.5 | Probably damaging | Damaging| Damaging	Low HDL-C
ABCA1	c.3343_3344delTC	p.S1115PfsX31	Frameshift	1	Yes	35 | NA | NA | NA	Low HDL-C
ABCA1	c.3544G>A	p.A1182T	Missense	1		20.9 | Benign | Tolerated | Damaging	Low HDL-C
ABCA1	c.4156G>A	p.E1386K	Missense	1		24.7 | Benign | Tolerated | Damaging	Low HDL-C
ABCA1	c.4430G>T	p.C1477F	Missense	1	Yes	34 | Probably damaging | Damaging | Damaging	Low HDL-C
ABCA1	c.5398A>C	p.N1800H	Missense	2	Yes	26.6 | Possibly damaging | Damaging | Damaging	Low HDL-C
ABCA1	IVS42+1G>A		Splicing	1		27.2 | NA | NA | NA	Low HDL-C
ABCA1	c.5672A>C	p.E1891A	Missense	1		25.2 | Probably damaging | Damaging |	Low HDL-C

						Damaging	
ABCA1	c.5774G>A	p.R1925Q	Missense	1		24.8 | Benign | Tolerated | Damaging	Low HDL-C
APOA1	c.85dupC	p.Q29PfsX29	Frameshift	2	Yes	28.3 | NA | NA | NA	Low HDL-C
APOA1	c.535delC	p.H179MfsX45	Frameshift	1		24.8 | Probably damaging | Damaging | Damaging	Low HDL-C
APOA1	c.566C>G	p.P189R	Missense	1	Yes	24.8 | Probably damaging | Damaging | Damaging	Low HDL-C
APOA1	c.718C>T	p.Q240X	Nonsense	1	Yes	35 | NA | NA | Damaging	Low HDL-C
CETP	c.164delC	p.S56AfsX11	Frameshift	1		36 | NA | NA | Damaging	High HDL-C
CETP	c.976C>T	p.Q326X	Nonsense	1	Yes	32 | NA | NA | NA	High HDL-C
LCAT	c.109_110delAC	p.T37AfsX3	Frameshift	1		37 | NA | NA | Damaging	Low HDL-C
LCAT	c.321C>A	p.Y107X	Nonsense	1	Yes	31 | Probably damaging | Tolerated | Damaging	Low HDL-C
LCAT	c.491G>A	p.R164H	Missense	2	Yes	27.8 | Probably damaging | Damaging | Damaging	Low HDL-C
LCAT	c.892A>C	p.T298P	Missense	1		31 | Probably damaging | Damaging | Damaging	Low HDL-C

LCAT	c.997G>A	p.V333M	Missense	1	Yes	31 | Probably damaging | Damaging | Damaging	Low HDL-C
LCAT	c.1039C>T	p.R347C	Missense	1	Yes	18.3 | Benign | Damaging | Tolerated | Damaging	Low HDL-C
LCAT	c.1244A>G	p.N415S	Missense	1	Yes	22.8 | Possibly damaging | Tolerated | Damaging	Low HDL-C
LIPC	c.193C>T	p.R65X	Nonsense	1		35 | NA | NA | Damaging	High HDL-C
LIPC	c.866C>T	p.S289F	Missense	1	Yes	25.5 | Probably damaging | Damaging | Damaging	High HDL-C
LIPC	c.1214C>T	p.T405M	Missense	3	Yes	24.6 | Probably damaging | Damaging | Damaging	High HDL-C
LIPC	c.1231G>C	p.G411R	Missense	1		27.5 | Probably damaging | Damaging | Damaging	High HDL-C
LIPG	IVS5+1G>T		Splicing	1		23.2 | NA | NA | Damaging	High HDL-C
LIPG	c.1187A>G	p.N396S	Missense	4		23.8 | Probably damaging | Tolerated | Damaging	High HDL-C
SCARB1	c.520C>T	p.R174C	Missense	1		32 | Probably damaging | Damaging | Damaging	High HDL-C

SCARB1	c.1258G>T	p.G420W	Missense	1		31 | Probably damaging | Damaging | Damaging	Low HDL-C
Secondary							
APOA5	c.944C>T	p.A315V	Missense	1	Yes	28 | Probably damaging | Damaging | Tolerated	Low HDL-C
APOC3	IVS2+1G>A		Splicing	2	Yes	25.1 | NA | NA | Damaging	High HDL-C
APOE	c.433G>C	p.G145R	Missense	1		28.4 | Probably damaging | Damaging | Tolerated	Low HDL-C
APOE	c.805C>G	p.R269G	Missense	2	Yes	25.6 | Benign | Damaging | Damaging	Low HDL-C
GPD1	c.208C>A	p.P70T	Missense	1		31 | Probably damaging | Damaging | Damaging	Low HDL-C
GPD1	c.760G>A	p.E254K	Missense	1		27.7 | Probably damaging | Damaging | Damaging	Low HDL-C
LMF1	c.1351C>T	p.R451W	Missense	2	Yes	24.9 | Probably damaging | Damaging | Tolerated	Low HDL-C
LMF1	c.1405G>A	p.A469T	Missense	2	Yes	29.7 | Probably damaging | Damaging | Damaging	Low HDL-C
LPL	c.644G>A	p.G215E	Missense	1	Yes	22 | Probably damaging | Tolerated |	Low HDL-C

LPL	c.701C>T	p.P234L	Missense	1	Yes	34 | Probably damaging | Damaging | Damaging	Low HDL-C

Abbreviations: NA = not available; del = deletion; dup = duplication; ext = extension; fs = frameshift; HDL-C = HDL cholesterol; HGMD = Human Gene Mutation Database.

[a] The order of prediction tool outcomes is: CADD PHRED Score, PolyPhen2, SIFT, and MutationTaster.

[b] Although this variant does not meet the described criteria for inclusion based on *in silico* prediction tools, it was included due to its sequence ontology of premature start codon.
